# Repeatable enhancement of healthcare data with social determinants of health

**DOI:** 10.3389/fdata.2022.894598

**Published:** 2022-08-01

**Authors:** Melody L. Greer, Cilia E. Zayas, Sudeepa Bhattacharyya

**Affiliations:** ^1^Department of Biomedical Informatics, University of Arkansas for Medical Sciences, Little Rock, AR, United States; ^2^Department of Biological Sciences, Arkansas Biosciences Institute, Arkansas State University, Jonesboro, AR, United States

**Keywords:** data quality, social determinants of health (SDOH), electronic health records (EHR), health informatics, healthcare, data curation

## Abstract

**Background:**

Social and behavioral aspects of our lives significantly impact our health, yet minimal social determinants of health (SDOH) data elements are collected in the healthcare system.

**Methods:**

In this proof-of-concept study we developed a repeatable SDOH enrichment and integration process to incorporate dynamically evolving SDOH domain concepts from consumers into clinical data. This process included SDOH mapping, linking compiled consumer data to patient records in Electronic Health Records, data quality analysis and preprocessing, and storage.

**Results:**

Consumer compilers data coverage ranged from ~90 to ~54% and the percentage match rate between compilers was between ~21 and 64%. Our preliminary analysis showed that apart from demographic factors, several SDOH factors like home-ownership, marital-status, presence of children, number of members per household, economic stability and education were significantly different between the COVID-19 positive and negative patient groups while estimated family-income and home market-value were not.

**Conclusion:**

Our preliminary analysis shows commercial consumer data can be a viable source of SDOH factor at an individual-level for clinical data thus providing a path for clinicians to improve patient treatment and care.

## Introduction

Socioeconomic and behavioral aspects of our lives significantly impact our health, yet minimal social determinants of health (SDOH) data elements are collected in the healthcare system. Information of this type is needed for quality healthcare research and patient care because it is associated with the full-spectrum of health outcomes from acute to chronic disorders. Studies indicate cancer (Alcaraz et al., 2020), cardiovascular disease (Tamura et al., 2019), dementia (Nicholas et al., 2021), mental health and substance-abuse disorders (Galea and Vlahov, 2002; Alegría et al., 2018), viral infection (Greer et al., 2021), and sleep (Grandner and Fernandez, 2021) are among a long list of health problems (Kivimäki et al., 2020) which are linked to social risk factors not frequently or consistently collected for patients. The combined effect of missing, inconsistent, or inaccurate data also leads to bias in machine learning, algorithms underlying clinical decision support, predictive analytics or other healthcare processes (Obermeyer et al., 2019; Cottrell et al., 2020; Seker et al., 2022). To avoid these problems as well as to gain rich insights from healthcare data we must be cognizant about diverse data collection, veracity and data-quality.

Electronic health records (EHR) are assembled from clinical, insurance and basic demographic information during the course of patient care. EHRs are real time, digital patient records containing, medical history, diagnoses, medications, treatment plans, immunizations dates, allergies, medical images, laboratory and test results. Modern EHR systems are built to go beyond standard clinical data collected in a healthcare facility and can include a broader view of a patient's care to provide a holistic assessment of a patient's health (Office of Disease Prevention Health Promotion, 2022a). Although there is growing interest in including SDOH data into EHR, the capture and management mechanisms for this process are uncertain (Ancker et al., 2018; Gold et al., 2018; Feldman et al., 2020). Current possibilities include (1) paper-based or digitally collected social needs screening before a patient receives care, (2) during a visit with a clinician, or (3) publicly available data sets that provide social context. All of these methods have challenges. Screening data that is incorporated into the EHR appears fragmented to users, increases the staff workload plus adds a data entry step where paper is used (Gold et al., 2018). And although clinicians felt SDOH information was valuable they are already pressed for time, and adding to their list of clerical tasks is often not practical or even possible (Tong et al., 2018). These barriers are likely the reason for the findings of Fraze et al. (2019) that social needs screening for food, housing, utilities, transportation, and experience with interpersonal violence was present in 24.4% of hospitals, and 15.6% of physician practices. SDOH data documented within unstructured EHR fields during a visit needs further study and will require natural language processing tools to be integrated into healthcare practice (Hatef et al., 2019). Publicly available data, whether in raw form (i.e., Census), or aggregated into measures [i.e., Social Vulnerability Index (SVI), Area Deprivation Index (ADI)] (Centers for Disease Control Prevention, 2015; Kind and Buckingham, 2018) has been valuable in studying the relationships between socioeconomic and patient health status (Johnson-Lawrence et al., 2017; Tung et al., 2018; Chamberlain et al., 2020). However, appending community-level data introduces issues with averaging. In their 2020 work on community-level and patient-level social risk data Cottrell et al. (2020) observed that community-level data misses some patients that patient-level data would not. Further complicating the issue, there are no currently accepted standard SDOH data elements (Cantor and Thorpe, 2018). This means that even in cases where social risk data is consistently collected it cannot be easily shared with other healthcare providers during transfers or referrals.

Compiled consumer data can address these issues. Consumer data is the trail of information that customers leave behind as a result of their purchases and internet usage. This data, collated from multiple sources, can comprise of personal information and are sourced from social media networks, marketing campaigns, customer service requests, call center communications, online browsing data, mobile applications, purchase history, preferences and many others. These data are constantly being collected and analyzed by companies as part of a broad customer relationship management strategy. Finance and marketing businesses have successfully used this data for over 20 years to find customers, understand their needs, and tailor financial products. Using these same strategies, healthcare researchers and providers can improve patient treatment and care. The consumer data includes individual-level SDOH data providing a holistic snapshot of an individual's lifestyle. It includes amongst others, income, education, lifestyle variables, language spoken, household size, smoking status, life events, hobbies, shopping activity etc. that are not available in the insurance claims data or majority of EHR data as shown in Table 1. A support system, for complex assessments (i.e., risk assessments) and calculations, is needed using information that helps to arrive at conclusions regarding patient's health risk and treatment. The development of an automated pipeline process for multisource healthcare data integration will provide this support. The presence of information integrated from multiple different streams of data will support tools for nurses, social workers, community health workers and patient navigators that supports decision making by providing the ability to consider multiple factors simultaneously for patients and clinicians. To begin the development of an automated pipeline process for multisource healthcare data integration we have conducted a pilot study integrating clinical and consumer information sources to evaluate multiple SDOH and clinical factors simultaneously.

**Table 1:** Consumer data and EHR data element categories.

**EHR**	**Consumer**
Basic demographics	Broad demographics
Clinical elements	Income bins
Diagnosis	Employment
Vital sign measures	Economic status bins
Inpatient and outpatient encounter	Education
Insurance	Insurance type
	Home owner status
	Vehicle owner status
	Lifestyle factors (i.e., sports, diet, smoking, and alcohol)
	Hobbies
	Neighborhood
	Weather

## Methods

**Figure 1:**
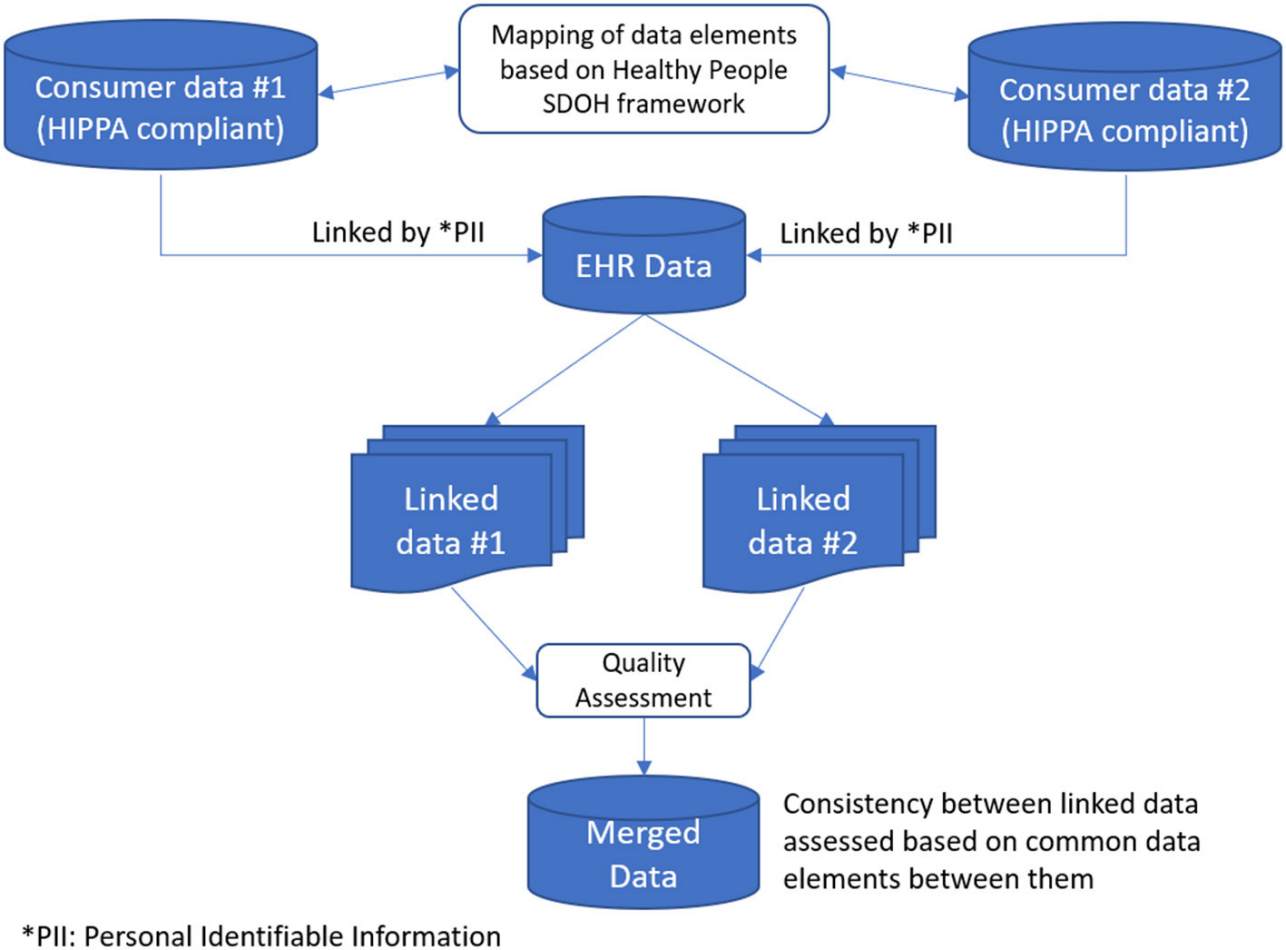
Workflow describing data integration of clinical and SDOH factors translated from consumer information sources.

Our goal was to prototype a repeatable clinical data enhancement process to incorporate compiled consumer data into SDOH domain concepts. This process included social risk factor mapping, linking compiled consumer data, data quality assessment, preprocessing, and storage. Consumer data does not currently line up one-to-one with factors identified as SDOH, so purchased data elements were mapped to concepts identified in social needs screening. Once the mapping was complete, the compiled consumer data elements were linked to the patient population and stored in a Microsoft SQL Server. The resulting data was then accessible using SQL queries, and the quality was evaluated for completeness, consistency, and timeliness or temporal alignment. Throughout SDOH enhancement, a security and data privacy layer overlaid the entire process with security features included in each step to ensure data privacy. Figure 1 depicts a snapshot of the entire process.

### Mapping

Various social determinants of health frameworks have been created to assist communities, healthcare professionals, and others in better identifying and managing an expansive range of factors influencing health outcomes (World Health Organization, 2010; Office of Disease Prevention Health Promotion, 2022a; Rural Health Information Hub, 2022). Our mapping strategy was developed based on the Healthy People (HP) 2030 SDOH Framework for this study (Office of Disease Prevention Health Promotion, 2022a,b). HP 2030 not only continued the HP initiative, which set national health targets for 2020 through 2030, but also designed a framework to organize SDOH into five domains: (1) economic stability, (2) education, (3) social and community context, (4) health and healthcare access, and (5) the neighborhood and built environment. HP 2030 outlined essential SDOH within each of these domains (Office of Disease Prevention Health Promotion, 2022b). Employment, food insecurity, housing instability, and poverty, for example, all fall under the domain of economic stability. We mapped as many elements as possible based on the HP 2030 framework from the two consumer data sources.

### Clinical data

Clinical data is collected in the EHR then selected elements are imported into the clinical data warehouse (AR-CDR) for research (University of Arkansas for Medical Sciences Translational Research Institute, 2022). The clinical data set used in our work was selected from the AR-CDR and contained over 54,000 individuals with comorbidities linked to high COVID-19 severity (e.g., diabetes and heart disease). The demographic characteristics included name, address, gender (55% female and 45% male), race (59% white, 34% Black or African-American, 6% were missing or unknown, and 1% were either American Indian, Alaskan native, native Hawaiian, Pacific Islander, or Asian), age (45% >65, 49% 31–65, and 6% <30). Clinical features were also collected for this study including diagnosis, procedures, inpatient status, and vital signs. The appended consumer data added 21 features from Compiler 1, and 842 features from Compiler 2 that were manually mapped to existing SDOH concepts as shown in Table 2.

**Table 2:** Data elements mapped from consumer databases to SDOH categories with coverage percentages listed for each individual compiler.

**SDOH domains**	**Element category**	**Compiler 1 coverage**	**Compiler 2 coverage**
**Economic stability**	**Employment**		
	• Occupation	48.23%	54.31%
	**Food insecurity**		
	• Health natural foods	Not reported	54.31%
	• Food and drink	Not reported	54.31%
	• Grocery	Not reported	54.31%
	**Housing instability**		
	• Homeowner/renter	98.98%	54.31%
	• Length of residence	98.98%	54.31%
	**Poverty**		
	• Estimated income	98.98%	54.31%
	• Net worth indicator	85.98%	54.31%
	• Economic stability indicator	85.98%	Not reported
	• Estimated discretionary income %	Not reported	54.31%
	• Estimated household debt level	Not reported	54.31%
	• Loan to value ratio	Not reported	54.31%
	• Public housing	Not reported	54.31%
**Education social and community context**	**Early childhood education and development**		
	• Education level	Not reported	54.31%
	**Enrollment in higher education**		
	• Occupation student	Not reported	54.31%
	• Presence of college graduate	Not reported	54.31%
	**High school graduation**		
	• Education level	Not reported	54.31%
	**Language and literacy**		
	• Country of origin	85.80%	Not reported
	• Hispanic language preference	85.80%	Not reported
	• Likes to read	Not reported	54.31%
	**Civic participation**		
	• Activism social issues	Not reported	54.31%
	• Community civic activities	Not reported	54.31%
	• Charitable volunteer	Not reported	54.31%
	• Registered voter indicator	Not reported	54.31%
	**Discrimination**	Not reported	Not reported
	**Incarceration**	Not reported	Not reported
	**Social cohesion**		
	• Community groups	Not reported	54.31%
	• Community and Family	Not reported	54.31%
	• Recreation	Not reported	54.31%
	• Sports	Not reported	54.31%
	• Travel family vacations	Not reported	54.31%
	• Caregiver in home	Not reported	48.89%
**Health and healthcare**	**Access to healthcare**		
	• Insurance	Not reported	54.31%
	• Percent healthcare uninsured	Not reported	54.31%
	• Prescription: number of drugs	Not reported	54.31%
	• Long term care insurance index	Not reported	37.46%
	• Medicare supplement insurance buyer index	Not reported	54.31%
	• Single service plan vision	Not reported	54.31%
	• Single service plan dental	Not reported	54.31%
	• Single service plan disability	Not reported	54.31%
	**Access to primary care**		
	• Health rank number of physicians	Not reported	48.89%
	• Health rank doctor visits	Not reported	48.89%
	**Health literacy**		
	• Reading cooking or culinary	Not reported	54.31%
	• Reading medical or health	Not reported	54.31%
	• Reading natural health remedies	Not reported	54.31%
**Neighborhood and built environment**	**Access to food that support healthy eating patterns**	Not reported	Not reported
	**Crime and violence**		
	• Concealed weapons	Not reported	54.31%
	**Environmental conditions**		
	• Census percent mobile homes	Not reported	54.31%
	• Census average number of automobiles	Not reported	54.31%
	• Digital neighborhoods	Not reported	54.31%
	**Quality of housing**		
	• Home market value, estimated	94.16%	54.31%
	• Home building repair	Not reported	54.31%

We requested electronic health records from the Clinical Data Repository (University of Arkansas for Medical Sciences Translational Research Institute, 2022) for all patients with chronic conditions (i.e., asthma, diabetes, heart disease, congestive heart failure, coronary artery disease, heart attack, and stroke), or contagious respiratory illness (i.e., influenza or COVID-19) between 2015 and 2020. All data received was stored on one of the following secure devices: institute supported controlled access server, institute supported password protected desktop computer, encrypted password protected laptop. The data used for linking social determinants information was name, address, DOB only. These demographics were transmitted to the selected data compiler vendors *via* SFTP. As per the requirement of the Health Insurance Portability and Accountability Act of 1996 (HIPAA) for protection of patient health information these 3rd party vendors signed a Business Associate Addendum (BAA) with our medical institution prior to accessing the patient identification. Following the addition of the SDOH, the data was de-identified to increase protection of the participants from any negative consequences in the event of a data breach. De-identification was accomplished by deleting full name and address and replacing them with a random identification number and RUCCA code (USDA Economic Research Service, 2020). The DOB was deleted and replaced with age. Data was stored in a secure database server behind a firewall.

### Non-clinical data integration and refresh process

Commercial data is updated monthly and is made up of hundreds of different sources, including consumer surveys, public records, purchase transactions, real estate data, offline and online buying behavior, and warranty information. Wherever possible, compilers compare values from multiple data sources to check accuracy for each element. Vendors that compile data for commercial purposes (i.e., marketing) were identified and interviewed, and then costs were negotiated. SDOH data points were appended by commercial data compilers or other external data sources using the identifiers, patient name, date of birth, and address. These processes occur entirely within a database system using fully HIPAA compliant vendors. All data was encrypted while in transit and immediately destroyed at the compiler location after the completion of the processing. Not all clinical data will be matched to existing consumer data during this process. This is the problem of coverage which refers to the number of patients who could be linked to compiled data. Coverage varies by commercial compiler, but the reasons for coverage variation may also be associated with varying aspects of the patient's lifestyle (e.g., people who use cash exclusively are less likely to have a substantial digital imprint in consumer databases).

Commercial compilers link information about consumers from multiple sources using individual or sets of identifying information like Social Security number, name, address, telephone number, and age or date of birth when available. This is done using proprietary matching algorithms that use statistical and rules-based methodologies. Often, elements are weighted within the algorithm based on their uniqueness within a larger population. In some cases, data elements are missing, making it impossible to link to the commercial data. In this work we have linked at the individual and household level in all cases where it was possible. Typically, studies have linked at the ZIP Code level using public data sources. In Table 2 we can see that Compiler 2 was unable to link almost 50% of the clinical data set while Compiler 1 reported values for fewer elements but had better match rates on those that were reported.

### Data quality analysis

Data quality is a constellation of factors essential for data collections. The quality of the linked clinical and commercial data (henceforth called merged-data) was evaluated for conformance, completeness, consistency, plausibility and temporal alignment (Kahn et al., 2016). As was mentioned above, accuracy, and consistency, were evaluated by compilers before data was purchased. After data was purchased and linked with EHR data we further measured consistency of common data elements between compilers from the different sources. In the merged-data there were 5 data elements that were common between the compilers. We matched the data records based on these 5 data elements to measure consistency between the compilers as an added level of data-quality assessment. Each of the data elements differed in categories or levels, between the compilers.

Our first step was to collapse the categories in each element into identical categories so that they could be compared for consistency. In some cases, the element values are inconsistent across clinical and compiler data sources as shown in Table 3. Although we preserved all of the values even if they were inconsistent this problem can be addressed by selecting values that occur most often across multiple sources, by giving preference to sources known to have higher quality data, or by using a gold standard such as direct patient contact. These techniques can be used individually or together depending on the needs of the end-user. For example, the data element “Home Market Value, Estimated” in compiler 1 mapped to “Home Value Range” in compiler 2. The categories of each these feature variables were however not the same. “Home Market Value, Estimated” in compiler 1 had the following 20 categories; “$1,000–24,999,” “$25,000–49,999,” “$50,000–74,999,” “$75,000–99,999,” “$100,000–124,999,” “$125,000–149,999,” “$150,000–174,999,” “$175,000–199,999,” “$200,000–224,999,” “$225,000–249,999,” “$250,000–274,999,” “$275,000–299,999,” “$300,000–349,999,” “$350,000–399,999,” “$400,000–449,999,” “$450,000–499,999,” “$500,000–749,999,” “$750,000–999,999,” “$1,000,000 Plus,” “NA”. The “Home Value Range” in compiler 2 had the following 18 categories; “Under $50 k,” “$50–100 k,” “$100–150 k,” “$150–200 k,” “$200–250 k,” “$250–300 k,” “$300–350 k,” “$350–400 k,” “$400–450 k,” “$450–500 k,” “$500–550 k,” “$550–600 k,” “$600–650 k,” “$650–700 k,” “NA,” “$700–750 K,” “$750 K +,” “Unknown”. In order to assess consistency we first collapsed the categories in each variable in an intuitive and meaningful fashion and made them identical. The new collapsed categories in both variables were: “Less 100 K,” “100–200 K,” “200–300 K,” “300–400 K,” 400–500 K,” “500 K plus”. After discarding the “NAs” or “Unknowns”, the consistency between the two variables were calculated.

**Table 3:** Percent matches between overlapping elements of the two compilers.

**Variable name**			
**Compiler 1**	**Compiler 2**	**No of categories**	**Percent match**
Home market value estimated	Home value range	6	55.68%
Marital status	Marital status	2	64.26%
Presence of children	Presence of children	2	41%
Income estimate household	Income range	9	20.85%

Missing data are a pervasive problem in any source of data also referred to as data “completeness”. Lack of complete data can significantly affect a study outcome by introducing unwanted bias. This is why it was important for us to obtain consumer marketing data from a diverse selection of sources to ensure that we have a collection of data that is complete and deep enough to provide meaningful information about majority of the study participants.

During preprocessing we also examined conformance and plausibility of the data elements, thus comparing the actual format of the data against the expected, and evaluating the feasibility of multiple existing values of the data elements. Measuring the persistence of the data was not possible in this work since to analyze changes in the data over time multiple batches of consumer data would be needed which was cost-prohibitive.

### Bias

Human bias exists. As we collect, analyze and take actions based on data our biases are perpetuated. This bias pervades healthcare in machine learning, decision support, operations and logistics planning in health systems. Applications to guide clinical practice are not exempt. Real-world clinical data is important for clinical decision-making but it has everyday biases imprinted within it and can preserve or even amplify health disparities. To address this issue requires detection and correction. Sensitive attributes (i.e., race, gender, etc.) are evaluated against classifications to first detect bias that may be present. Once uncovered biased data can be rebalanced using class labels. While this method is not foolproof, biases that are not expected may remain hidden, and it does allow for the mitigation of known issues and for the discovery of unknown issues.

### Data analysis

After the data was linked and preprocessed we explored the data statistical software packages that were linked to the SQL Server hosting the merged-data. We determined the summary statistics of a subset of the variables, from both EHR and compiled SDOH sources, that characterized the patients. We are also currently in the process of building classifiers that can predict disease risk based on patients' clinical, demographic and SDOH factors.

## Results

### Mapping and linking consumer data with EHR at an individual-level

Table 1 lists SDOH domains, elements, and coverage percentages from two consumer data databases that were used in our study and named here as compiler 1 and compiler 2. There were 55,422 patients in the initial set of EHR records. After linking with two commercial compilers, 30,895 and 54,880 patients with SDOH data remained, respectively. As the table shows, compiler 1 had fewer mapped data elements compared to compiler 2. However, compiler 1 had, on an average, ~90% coverage on the mapped data elements while compiler 2 had many more elements mapped but the coverage was much lower, ~54% on an average. Thus, consumer data collection from at least two sources ensured that all categories of SDOH domains, based on Healthy People 2020 SDOH Framework were covered in our merged-data (Office of Disease Prevention Health Promotion, 2022a). The connection between unlinked patients needs further study to determine what they have in common aside from a minimal individual digital footprint.

### Data quality assessment and preprocessing

To measure consistency between the compiler data collected from different sources we matched the data records based on elements that were common between the compilers as part of data-quality assessment. Table 3 shows the randomly picked data elements and their percent matches. The percent matches of compiler 2 ranged between ~21 and 64% with compiler 1. This underscores the importance of data collected from multiple, trusted and standardized sources.

### Bias

In healthcare as in other areas existing data is sometimes used to power algorithmic prediction. However, when predictions are based on biased information we can proliferate existing biases (Seyyed-Kalantari et al., 2021). Comorbidity indices are common clinical data used for risk adjustment based on patient characteristics (Alonso-Morán et al., 2015) and comorbidity rises with age (Boersma et al., 2020). Yet rural populations in Arkansas are less likely to be diagnosed with multiple chronic conditions as they age than their urban counterparts (Seker et al., 2022). To study this problem of bias in healthcare data, we have measured the amount of bias due to home location and preprocessed a real-world data set of patients with chronic conditions from geographically disparate locations. Bias in data that will be used for modeling can be addressed at one of three different time points: (1) before it is used for modeling, (2) during the modeling process, or (3) after modeling is complete. We have chosen to correct bias as a preprocess because it prepares the data for downstream modeling and does not need to be repeated for each new model. Irrespective of the timepoint when bias processing occurs, it must not damage the integrity of the data and thereby negatively impact modeling. To monitor this potentiality, we also tested addressing bias by removing the biased data element, the location of the home residence from models. Each of these tests produced similar AUC results to the original model constructed with unaltered biased data indicating that data preprocessed for bias still performs well when used for modeling as described previously (Seker et al., 2022).

### Preliminary analysis and visualization of the merged data

**Figure 2:**
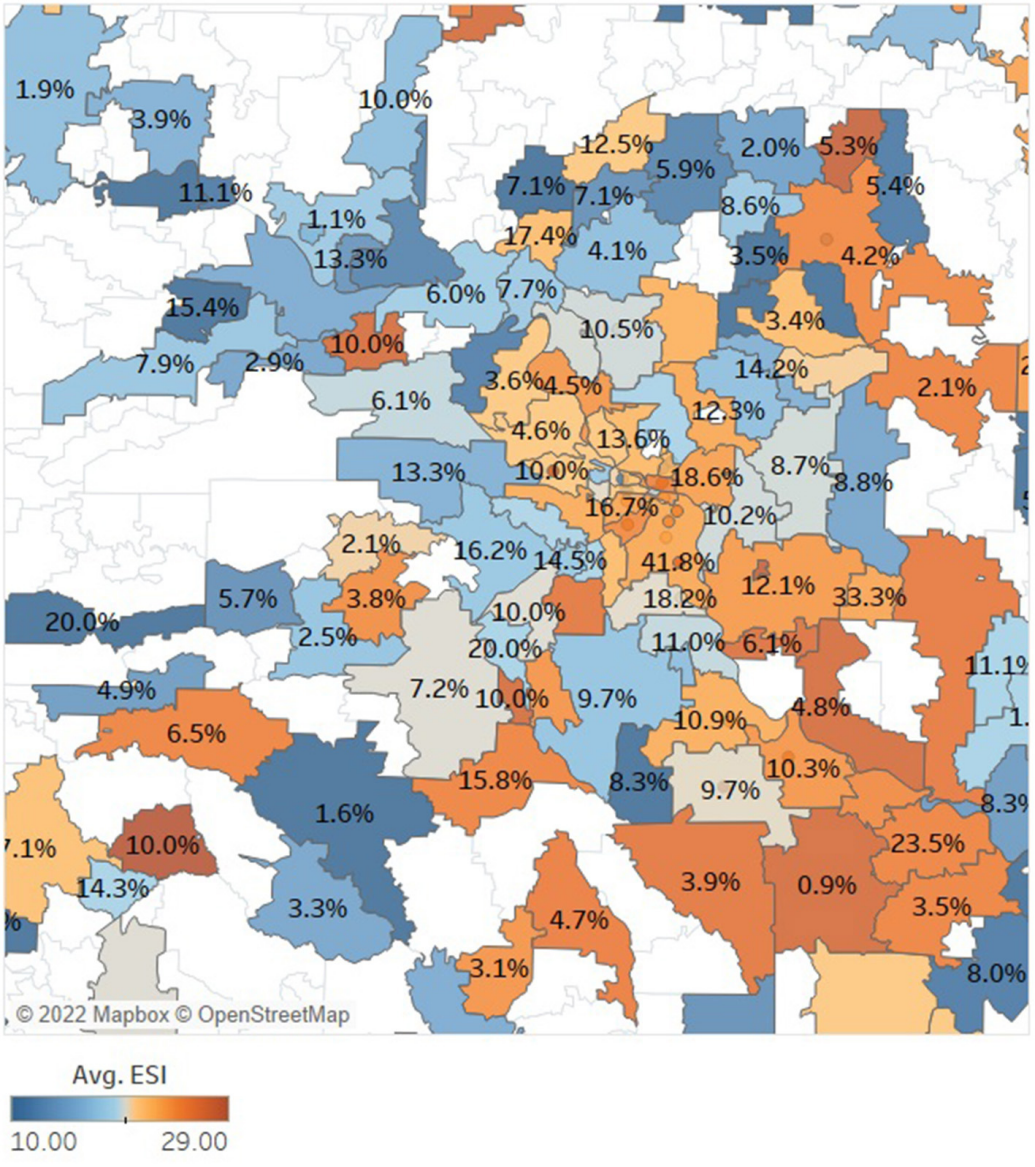
This map of central Arkansas, built using our final merged clinical and consumer data of patients with chronic conditions or respiratory illness, shows that lower economic stability (indicated in orange) trends with higher instances of COVID-19 positivity (indicated in percentage label) but that was not true for all zip codes as there were other SDOH confounders. White zip codes indicate no data was included for the area.

The SDOH data merged with EHR provided insights into the social risk factors of disease both at patient level as well as the population level. We first explored the disparities in demographics and SDOH factors between the 55,422 patients who were COVID-19 positive compared to those who were not. Table 4 provides a summary of a subset of patient characteristics that were compared between these two groups. Our preliminary analysis showed that apart from demographic factors, several SDOH factors like home-ownership, marital-status, presence of children, number of members per household, Economic Stability Indicator (ESI) and education were significantly different between the two patient groups while estimated family-income and home market-value were not. Figure 2 shows a map constructed with the merged clinical and consumer data. In this figure the top COVID-affected zip codes in Arkansas are overlaid on a heatmap image of average ESI of those zip codes. ESI is a proprietary consumer data element provided by compiler 1 and shown in Tables 2, 4. It is commonly used in marketing which is constructed to function like a credit score but generated without credit data. The darker orange counties reflected patients from our data that were less economically stable and also had higher percentages of COVID-19 positive patients.

**Table 4:** Summary statistics of demographics and individual-level SDOH factors in the merged data with COVID-19 status as indicated by International Classification of Diseases (ICD) codes.

		**COVID-19 positive**		
**Characteristics**	***N***	**No, *N* = 54,330^a^**	**Yes, *N* = 1,092^a^**	***p*-value^b^**
**Gender**	55,422			0.032
F		29,611 (55%)	638 (58%)	
M		24,706 (45%)	454 (42%)	
U		13 (<0.1%)	0 (0%)	
**Age**	55,422	62 (18)	46 (18)	<0.001
**Race**	46,869			<0.001
African American		10,220 (22%)	298 (32%)	
Asian		551 (1.2%)	25 (2.7%)	
Hispanic		1,445 (3.1%)	110 (12%)	
White/other		33,726 (73%)	494 (53%)	
**Home owner renter**	54,068			<0.001
Home owner		34,558 (65%)	605 (56%)	
Renter		18,420 (35%)	485 (44%)	
**Marital status**	54,068			<0.001
Married		26,140 (49%)	391 (36%)	
Single		26,838 (51%)	699 (64%)	
**Has children**	54,068	14,826 (28%)	377 (35%)	<0.001
**Member household**	54,068			<0.001
1		20,473 (39%)	487 (45%)	
2		15,603 (29%)	224 (21%)	
3		7,772 (15%)	173 (16%)	
4		5,201 (9.8%)	112 (10%)	
5		3,305 (6.2%)	71 (6.5%)	
6		393 (0.7%)	13 (1.2%)	
7		145 (0.3%)	3 (0.3%)	
8		61 (0.1%)	6 (0.6%)	
8 Plus		25 (<0.1%)	1 (<0.1%)	
**Estimated family income**	34,792			0.4
<30 K		0 (0%)	0 (0%)	
$30–50 K		14,070 (41%)	271 (43%)	
$50–75 K		9,228 (27%)	165 (26%)	
$75–100 K		4,448 (13%)	90 (14%)	
$100–125 K		2,567 (7.5%)	36 (5.7%)	
$125 K plus		3,848 (11%)	69 (11%)	
**Estimated home market value**	51,438			0.9
<100 K		19,231 (38%)	405 (39%)	
100–200 K		21,183 (42%)	434 (42%)	
200–300 K		5,719 (11%)	124 (12%)	
300–400 K		2,153 (4.3%)	39 (3.7%)	
400–500 K		878 (1.7%)	20 (1.9%)	
500 K plus		1,230 (2.4%)	22 (2.1%)	
**Economic stability indicator**	37,135			<0.001
10–15		7,126 (20%)	117 (15%)	
16–20		7,696 (21%)	118 (15%)	
21–25		10,940 (30%)	226 (28%)	
26–30		10,573 (29%)	339 (42%)	
**Education**	34,295			<0.001
Attended vocational/technical		361 (1.1%)	5 (0.7%)	
Completed college		11,392 (34%)	187 (28%)	
Completed graduate school		4,008 (12%)	45 (6.6%)	
Completed high school		17,857 (53%)	440 (65%)	

^a^*Statistics presented: n (%); mean (SD)*.

^b^*Statistical tests performed: chi-square test of independence; Wilcoxon rank-sum test*.

## Discussion

In this proof-of-concept study our main objective was to evaluate the viability of consumer marketing data, purchased from 3rd party HIPPA-compliant vendors, as a source of SDOH factors that are largely missing from the EHR data. We purchased in-depth patient-level consumer data from two different vendors, mapped a wide array of data elements to the 5 broad SDOH domains as defined by the Healthy People 2030 SDOH framework, linked the consumer data to EHR patient level data, stored the data in a SQL server linked with several statistical and data exploration tools, evaluated data quality and preprocessed the data, and lastly completed a preliminary analysis of a subset of the SDOH elements that characterized the patients. To our knowledge this is the first study to explore the viability of consumer marketing data as a source of patient-level SDOH data.

With recent upsurge in research solidifying the significant relationship between SDOH and population health, an increasing number of healthcare stakeholders are exploring the use of public databases for community-level information in order to identify those patients that are most vulnerable to SDOH. For example, census tracts data have been used to identify areas associated with socio-economic risks and poor health outcomes (Liaw et al., 2018). But a recent study by Cottrell et al. (2020) showed that only about 48% of the times community-level data can accurately identify social risks at the patient level. Thus, healthcare decisions on individual patients based on community-level data may fall short on providing adequate care to a significant number of patients. This may give rise to the problem of “ecologic fallacy” where incorrect assumptions can be made about a patient based on aggregate-level information from community-level data (Garg et al., 2016; Cottrell et al., 2020). In this study we have attempted to address this problem by partnering with companies/vendors that are honed consumer market researchers.

We have developed a repeatable process to incorporate commercially compiled data into EHR data. The added value has been demonstrated based on a published paper (Greer et al., 2021). We have also identified opportunities in data quality research areas that need further study as part of this work. The curated data are being used to support several healthcare analytics applications, including descriptive analytics, and predictive modeling. During this work, we have developed the first mapping scheme of commercial data elements with SDOH elements. This is a fascinating aspect of this work because the healthcare community has not reached a consensus on a standard set of social determinants of health concepts demanding that this process be agile and flexible.

While building an enrichment process for EHR data we had to address important issues related to temporal alignment, data dictionary and coverage, legal requirements, and security requirements. Initially, the legal, research and business processes required were complex and time-consuming. Patient data must be kept private and secure at all times, and all parties must be bound by a contract to minimize the possibility of a data breach. Fortunately, once contracts and transfer processes are in place, they remain active and available for repeated consumer data collection. This is important because continued collection will be necessary. Compiled data becomes stale over time, and EHR data is collected only at the time of each encounter. Aligning these time windows is necessary for elements that must be current while is less critical for elements that are more likely to remain stable over time. As the process iterates and newly compiled data is integrated into the EHR data, the data dictionary must also be updated. We discovered that the data dictionaries provided by compilers vary in quality and detail. In addition, compilers are continuously adding, removing, and updating elements resulting in multiple versions of the dictionary documentation. Integrating data from these dynamic systems also impacted the mapping of compiled elements onto SDOH concepts, resulting in a mapping component for each iteration. If kept current, the SDOH mapping will require minimal effort to maintain. Throughout all of these components, it was also necessary to tackle practical information technology issues such as storage, tools, permissions, and access which will need to be customized to each institution.

Our study had several limitations. Due to budgetary constraints we restricted our SDOH data sources to two different vendors only. One had more mapped data elements while the other had more coverage, thus highlighting the need for data collection from multiple, trust worthy and reputable vendors with standardized methods of data collection. There were significant missing data in each data set. Also, among the overlapping data elements, the concordance between the data was not very high which underscores the need for good quality data sources.

In conclusion, we have developed a repeatable SDOH enhancement process to incorporate dynamically evolving SDOH domain concepts from consumers into clinical data. The literature provides early and rapidly growing evidence that integrating individual-level SDOH into EHRs can assist in risk assessment and predicting healthcare utilization and health outcomes, which further motivates efforts to collect and standardize patient-level SDOH information. This study highlights one potential means to incorporate individual-level patient data into EHR, thus opening up possibilities for predictive analytics and enhanced solutions for providers, payers and healthcare organizations to enable them to address the social needs of patients.

## Data availability statement

The datasets presented in this article are not readily available because it contains clinical information. Requests to access the datasets should be directed to mlgreer@uams.edu.

## Ethics statement

The studies involving human participants were reviewed and approved by University of Arkansas for Medical Sciences IRB. Written informed consent for participation was not required for this study in accordance with the national legislation and the institutional requirements.

## Author contributions

MG developed the concept. MG and SB worked on concept development analysis and manuscript writing. CZ performed SDOH mapping and writing. All authors contributed to the article and approved the submitted version.

## Funding

The research reported in this publication was supported by the National Center For Advancing Translational Sciences of the National Institutes of Health under Award Numbers TL1 TR003109 and UL1 TR003107.

## Conflict of interest

The authors declare that the research was conducted in the absence of any commercial or financial relationships that could be construed as a potential conflict of interest.

## Publisher's note

All claims expressed in this article are solely those of the authors and do not necessarily represent those of their affiliated organizations, or those of the publisher, the editors and the reviewers. Any product that may be evaluated in this article, or claim that may be made by its manufacturer, is not guaranteed or endorsed by the publisher.

## Author disclaimer

The content is solely the responsibility of the authors and does not necessarily represent the official views of the National Institutes of Health.
